# Multi‐Level Approaches for Assessing Molecular and Physiological Traits of Drought and Heat Stress Tolerance in Plant Reproductive Development

**DOI:** 10.1111/ppl.70760

**Published:** 2026-02-01

**Authors:** Christos Bazakos, Marija Vidović, Aleksandra Radanović, Ariola Bacu, Silvana Francesca, Maria Manuela Rigano

**Affiliations:** ^1^ Institute of Plant Breeding and Genetic Resources—ELGO Dimitra Thessaloniki Greece; ^2^ Institute of Molecular Genetics and Genetic Engineering University of Belgrade Belgrade Serbia; ^3^ Institute of Field and Vegetable Crops National Institute of the Republic of Serbia Novi Sad Serbia; ^4^ Department of Biotechnology University of Tirana Tirana Albania; ^5^ Department of Agricultural Sciences University of Naples Federico II Naples Italy

**Keywords:** combined abiotic stress, heat stress, multi‐omics, stress resilience

## Abstract

Abiotic stress, particularly heat and drought, significantly impacts plant reproductive development, threatening crop productivity and food security. Understanding stress tolerance mechanisms requires a multi‐level approach that integrates physiological, biochemical, and molecular traits in different experimental settings. This review explores key methodologies for assessing resilience to single and combined abiotic stress in reproductive tissues, from growth chamber experiments to greenhouse and field trials. Essential physiological and biochemical traits indicative of stress responses are highlighted alongside molecular pathways that provide deeper insights into adaptation to drought and heat stress. The use of multi‐omics techniques, including transcriptomics, proteomics, and metabolomics, as powerful tools for identifying novel stress‐associated traits is discussed, with an emphasis on the integration of these techniques into a holistic framework, which also incorporates single‐cell approaches. Finally, we address the limitations of the current methodologies and propose future research directions to improve stress resilience assessment in plant reproductive development.

## 1 Introduction

Projections indicate that global temperatures could increase by 1.8°C–4.0°C by the end of the century (Scafetta 2024), and the resulting heat stress is already causing crop yield losses, with annual losses reaching up to $50 billion worldwide (Qian et al. 2025). While these issues raise great concern, we know that, in general, plants can maintain their vegetative growth and development over a relatively wide range of temperatures. The survival thermal range goes from −10°C in woody trees of regions such as Alaska, northern Canada, Europe, and Asia up to +60°C in plants from arid regions such as cacti and agaves (Nievola et al. 2017). Plants have evolved complex mechanisms to cope with daily changes in the environment thanks to mechanisms classified as avoidance and tolerance (Mishra et al. 2021) or avoidance, escape, and tolerance (Bacu et al. 2024). Avoidance includes various morphological adaptations or accumulation of stress‐associated proteins, while tolerance involves alterations in the expression of genes encoding ion transporters, transcription factors, or enzymes involved in the biosynthesis of osmoprotectants and antioxidants.

Heat stress can alter multiple aspects of cellular physiology (membrane fluidity, nucleic acid and protein structures, metabolite and osmolyte concentrations) (Chinnusamy et al. 2007; Zinn et al. 2010), accelerate production of reactive oxygen species (ROS), inhibit photosynthesis, damage the oxygen‐evolving complex of photosystem II (PSII), reduce Rubisco activity (Law and Crafts‐Brandner 1999), and cause disorganization of the thylakoid membranes (Gounaris et al. 1984). The developmental stage at which a plant is exposed to heat stress strongly affects the overall response. Heat stress during reproductive stages is more detrimental compared to stress during vegetative stages in both annual and woody perennial crops (Hussain et al. 2019; Jagadish et al. 2021). During the transition from the vegetative to the reproductive stage, the environmental conditions affect photosynthetic efficiency, canopy development, and fruiting initiation (Talwar et al. 2022). The accelerated rise of air temperatures has negative effects on flower bud formation, flowering, fruit ripening, and seed germination, resulting in lower seed yields and quality (Raza et al. 2019; Yadav et al. 2022). Flowers and their constituent parts are highly sensitive to temperature. Even slight increases in temperature above the optimum during the gametophytic phase can impair reproduction by affecting micro‐ and macrosporogenesis, reducing pollen number and viability and increasing embryo abortion (Hedhly 2011; Tushabe et al. 2023; Zinn et al. 2010). Male gametophyte development is particularly heat‐sensitive, leading to reduced pollen vigor and viability, pollen sterility, inhibited pollen tube growth, and decreased pollen germination (Koti et al. 2005; Talwar et al. 2022). Heat stress also enhances ROS accumulation, which causes oxidative damage, membrane disruption, and deformed pollen grains (Djanaguiraman et al. 2018; Milić et al. 2023). Elevated temperatures further compromise pollen–stigma interactions by reducing stigma receptivity, ovule life span, and pollen adherence, while also altering stigma and style morphology, thereby limiting pollination efficiency (Chabert et al. 2018; Montalt et al. 2019).

Plants frequently encounter combinations of stress factors (Bokszczanin et al. 2013). Indeed, heat and drought stress often co‐occur, intensifying each other's impact on plants (Qian et al. 2025) and resulting in abnormal anther development and significantly reduced pollen viability, affecting pollination efficiency (Katano et al. 2020; Su et al. 2013). Drought also affects female reproductive organs by limiting the expansion of embryo sacs, leading to degeneration and malformation, and impairing ovule development (Zahra et al. 2021). Moreover, drought is an obstacle to pollination and fertilization. Dehydration and structural abnormalities in stigmas reduce pollen adhesion, thereby decreasing pollination success and seed yields (Fábián et al. 2019).

The combined drought and heat stress on crop reproduction represents a complex systemic challenge, involving interactions among crop species, reproductive organs, key genes, and physiological mechanisms. Building on the evidence that both stresses strongly impair reproductive structures, further exploration of heat‐ and drought‐responsive genes, particularly those associated with reproductive stages, is crucial. Future research should focus on crop reproductive processes under combined stress, broaden the scope to include economically important crops and female reproductive organs, and establish high‐throughput phenotyping systems to generate accurate datasets (Qian et al. 2025). As emphasized by Jagadish et al. (2021), alternative methods are needed to impose and quantify stress in tissues and developmental stages that are less accessible (e.g., floral meristem), while integrating recovery dynamics into breeding programs. Despite its importance, post‐stress recovery remains underexplored and is rarely incorporated into efforts to improve stress tolerance. Also, complementary approaches such as artificial pollination, precision agriculture tools (e.g., remote sensing technologies and climate models for heat risk prediction), and the application of small‐molecule compounds (e.g., plant growth regulators during high‐temperature periods) could help enhance plant tolerance (Hoffmann and Sgrò 2011; Khan et al. 2020).

In the following sections of this review, key methodologies for assessing resilience to single and combined drought and heat stress will be discussed, ranging from growth chamber experiments to greenhouse and field trials.

## 2 From Growth Chamber to Greenhouse and Open Field: Investigating Responses to Drought and Heat Stress

The response of crops to heat stress has been extensively investigated over the past two decades; however, designing appropriate stress experiments remains a complex task as the experimental setup can strongly influence outcomes. For example, it has been shown that the effect of heat on grain yield in winter wheat is substantially dependent on the temperature measurement point (ear, leaf, canopy or ambient air), the method of heating (ambient air heating in growth chambers or direct heating of ears by infrared heaters), and the soil substrate used (Rezaei et al. 2018). Several protocols for drought studies have also been established under both controlled conditions and open fields (Korwin Krukowski et al. 2020; Moshelion et al. 2024; Osmolovskaya et al. 2018). Often, drought research methods include a soil pot water control method, in which researchers use a weighing method or soil moisture meter to control the soil moisture content. Alternatively, osmotic substances such as mannitol and polyethylene glycol (PEG 6000 or PEG 8000) are applied to simulate drought, collectively referred to as “osmotic stress” (Wang et al. 2024). PEG can be combined with a nutrient solution under hydroponic conditions or used with sand culture to simulate drought. It should be emphasized, however, that PEG‐induced osmotic stress does not fully mimic soil drought. While PEG lowers the osmotic potential and restricts water uptake, it does not reproduce soil hydraulic properties, root–soil interactions, or nutrient limitations typical of field drought. Moreover, high PEG concentrations can reduce oxygen diffusion and introduce toxicity effects, which may lead to misinterpretation if results are extrapolated directly to natural drought conditions.

Heat stress experiments are generally more difficult to establish. Stress can be imposed at different temporal scales, intensities, and durations using diverse facilities, including leaf and plant chambers, glasshouses, field‐based tents, radiant heaters, and naturally hot summer months (Jagadish et al. 2021). The first thing we should consider when setting up an experiment to analyse heat stress is the aim of the study. Indeed, to perform molecular studies and follow the precise expression dynamics of different genes, experiments could be conducted in controlled rooms under steady light, temperature, and humidity, parameters that normally fluctuate in open fields. On the contrary, if the aim is to identify tolerant genotypes, a field experiment to quantify crop performances and yield is preferable (Korwin Krukowski et al. 2020). In field trials, one of the biggest problems is the complexity of the interactions between plants, soil, geography, climate and weather conditions, and other potential confounding factors, including biotic stresses. To address the challenges posed by field conditions, multi‐location and multi‐seasonal experiments are usually performed (Francesca et al. 2024; Horváth et al. 2024).

Before starting heat stress experiments, some key considerations should be included: selecting the stress parameters to be measured, the developmental stage, and the plant organ of interest. It should be considered that the early seedling stage and reproductive period are among the most heat‐sensitive stages (Jagadish et al. 2021). Another complication is that, especially with sandy soil substrates, heat stress studies often involve concurrent drought, which amplifies the heat effect by reducing transpiration and photosynthesis through stomatal closure (Kumari et al. 2024; Qian et al. 2025). The duration of stress treatment is also critical. Many studies apply very high temperatures (45°C–50°C) for short periods (30 min to 3 h), inducing a classical heat shock response, including the rapid synthesis of HSP (Jagadish et al. 2021). While such experiments provide insight into tolerance mechanisms, they do not reflect chronic stress conditions. Thus, the evaluation of climate‐resilient crops should consider the impact of heat waves and longer periods of elevated temperatures (Bollier et al. 2023). Heat wave studies typically expose plants to temperatures 5°C–10°C above optimum growth conditions for several hours to days, sometimes repeating the stress after recovery (Jagadish et al. 2021). Chronic heat stress experiments instead expose plants to moderately elevated temperatures (5°C–10°C rise in air temperature) for one or more weeks during key developmental stages (i.e., flowering). For example, short episodes of high temperature (27°C–31°C) around anthesis significantly reduced grain yield in cereals (Rezaei et al. 2018), while exposure of tomato plants to chronic heat stress (35°C/25°C, day/night) for three weeks enabled the identification of tolerant genotypes through analysis of microsporogenesis and pollen germination (Bollier et al. 2023). Given that global warming will increase the frequency and intensity of extreme events with stronger impacts on crops, future studies should place greater emphasis on simulating heat waves and chronic heat stress. A stronger integration of knowledge across heat shock, heat wave, and long‐term warming studies will help us identify and develop genotypes with higher resilience in their target environments (Jagadish et al. 2021).

## 3 Morphophysiological Traits Indicative of Response to Drought and Heat Stress

**FIGURE 1:**
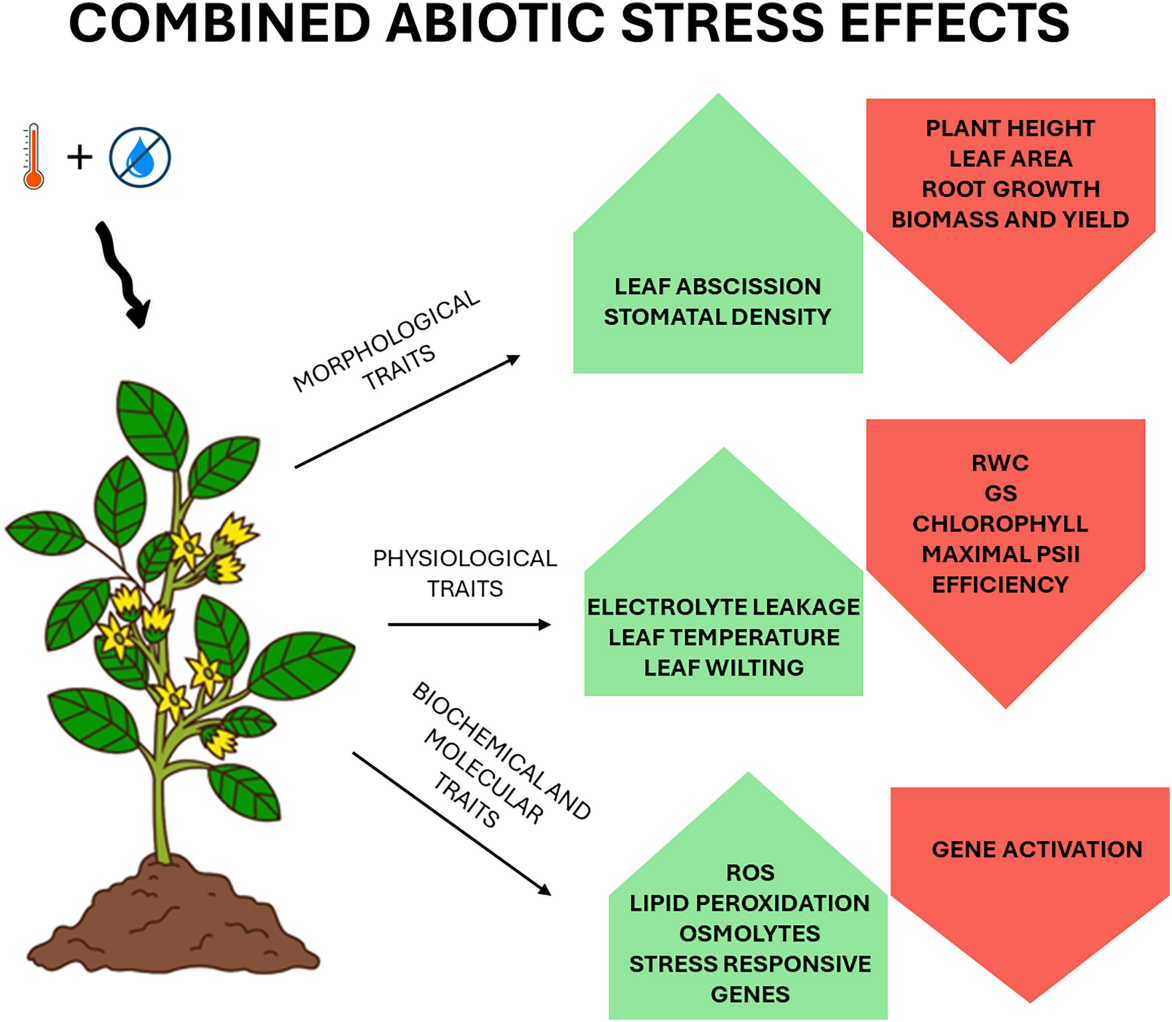
Effects of combined drought and heat stress on plant performance. Combined drought and heat stress impacts multiple plant traits causing morphological changes, physiological alterations, and molecular responses.

Morphological traits associated with both vegetative growth and reproductive development serve as key indicators for monitoring abiotic stress in plants (Figure 1). Heat and drought stress typically reduce plant height and leaf dimensions (length, width, and thickness), reflecting adaptive mechanisms to minimize water loss and thermal load (Jiang et al. 2025). Alterations in leaf morphology, such as increased leaf rolling or curling, are also observable responses to elevated temperatures that help reduce transpiration and photodamage. During the reproductive phase, heat stress may either delay or accelerate flowering depending on species and genotype, with significant modifications in anthesis timing that directly influence reproductive success (Sinha et al. 2022). Heat stress further alters floral morphology, causing decreased pollen viability, abnormal anther dehiscence, and reduced ovule fertilization rates, compromising seed set and yield (Liu et al. 2022). Floral organ abortion and structural changes in inflorescences are also frequent (Qian et al. 2025). Monitoring these morphological traits provides valuable phenotypic markers for screening heat tolerance. Recent advancements in noninvasive phenotyping technologies facilitate high‐throughput quantification of these traits, supporting the identification of heat‐resilient genotypes. Water availability critically interacts with heat stress, often worsening its effects. Water scarcity limits plants' ability to cool through transpiration, increasing heat damage. Monitoring morphological changes under combined heat and water stress provides a better understanding of plant resilience.

Photosynthetic performance is among the most sensitive and widely used indicators of combined stress (Figure 1). Chlorophyll fluorescence imaging has become a vital non‐invasive technique for evaluating spatial and temporal variations in photosynthetic activity. This approach provides high‐resolution mapping of photosynthetic parameters across leaf surfaces, revealing heterogeneity in stress responses that conventional point measurements may overlook (Moustaka and Moustakas 2023). The PSII quantum yield, particularly the ratio of variable to maximum chlorophyll fluorescence (Fv/Fm), is a primary diagnostic measure of photosynthetic efficiency during stress (Kalaji et al. 2016). A reduction in Fv/Fm values indicates photoinhibition and damage to the photosynthetic machinery, particularly the oxygen‐evolving complex and thylakoid membrane stability. For example, wheat exposed to temperatures exceeding 35°C exhibited pronounced Fv/Fm reductions, correlating with diminished photosynthetic capacity and substantial yield losses (Sommer et al. 2023). The sensitivity of Fv/Fm to heat stress has been leveraged to identify tolerant cultivars. Studies on tomato revealed that cultivars showing minimal Fv/Fm declines under thermal stress were more heat‐tolerant, maintaining photosynthetic activity and growth more effectively (Bita and Gerats 2013). Similarly, Arief et al. (2023) utilized variations in Fv/Fm values to detect differences between control treatments, drought, heat stress, and combinations of both. Beyond Fv/Fm, additional chlorophyll fluorescence parameters such as non‐photochemical quenching (NPQ) and electron transport rate (ETR) offer insights into protective mechanisms employed by plants under combined stress (Havaux 2014). NPQ, which quantifies a plant's ability to dissipate excess absorbed light energy as heat, acts as a vital photoprotective mechanism to prevent photoinhibition and oxidative damage (Murchie and Lawson 2013). Recent investigations in wheat and maize have shown that heat‐tolerant cultivars exhibit a rapid and sustained NPQ response, associated with better preservation of PSII integrity under elevated temperatures (Zuo 2025). In addition to NPQ, steady‐state chlorophyll fluorescence yield (Fs) and the efficiency of open PSII reaction centers (Fv′/Fm′) provide complementary data on the functionality of the photosynthetic system. These parameters help differentiate between reversible photoprotective downregulation and irreversible photodamage. For example, in soybeans, transient reductions in Fv’/Fm′ during brief periods of high temperature were reversible when plants were returned to optimal temperatures. However, prolonged exposure to high temperatures led to permanent reductions, indicating damage to PSII (Wang et al. 2022).

Cellular membrane stability represents another important trait for assessing stress tolerance. Membrane thermostability is commonly assessed through electrolyte leakage assays, by measuring the release of intracellular ions into surrounding solutions, with higher conductivity values indicating greater membrane disruption (Sommer et al. 2023). Tolerant plants typically exhibit lower electrolyte leakage rates and possess higher critical temperatures for membrane dysfunction. The lipid composition and fluidity, especially the balance between saturated and unsaturated fatty acids, greatly influence membrane stability. Heat‐resilient plant species generally have higher proportions of saturated lipids and sterols that increase membrane rigidity at elevated temperatures (Niu and Xiang 2018).

Heat and drought stress also profoundly influence plant gas exchange by impacting stomatal conductance and biochemical pathways involved in photosynthesis and transpiration. Many crops, such as wheat, show reductions in net photosynthesis and stomatal conductance under combined stress, leading to diminished carbon assimilation (Li et al. 2023). Conversely, some species maintain or increase stomatal conductance in response to heat to enhance transpirational cooling; this mechanism, observed in wheat and sorghum, can lead to rapid water loss and does not invariably prevent stress. In rice, acclimation enables sustained net CO_2_ assimilation under heat only if the leaves develop during prolonged elevated temperatures; otherwise, both gas exchange and growth deteriorate under high temperature (Hüve et al. 2011).

Biochemically, stress can cause Rubisco deactivation and elevated photorespiration rates, restricting photosynthetic capacity in species such as rice and wheat (Lal et al. 2022). Drought significantly reduces Rubisco activity in plants through multiple mechanisms, including decreased enzyme synthesis, reduced activation state due to limited ATP and NADPH availability, and conformational changes in the enzyme structure. Extreme heat destabilizes photosynthesis through membrane damage and increases ROS production, which impairs physiological recovery and threatens long‐term function (Hüve et al. 2011). These effects differ among species and cultivars, with certain rice and maize varieties showing better heat acclimation than wheat (Lal et al. 2022).

Recent advances in remote sensing technologies have markedly improved the monitoring of stress indicators across large spatial scales. Thermal infrared imaging enables the precise measurement of canopy temperature and its deviation from ambient air, serving as an indicator of evaporative cooling capacity via transpiration (Yang et al. 2025). Hyperspectral imaging provides comprehensive spectral data that detect changes in chlorophyll content, plant water status, and biochemical compounds associated with stress responses (Poobalasubramanian et al. 2022). Furthermore, fluorescence‐based remote sensing approaches, such as solar‐induced chlorophyll fluorescence, enable high‐resolution, spatially explicit monitoring of photosynthetic performance and stress‐induced physiological changes across agricultural and natural ecosystems, offering critical insight into plant responses to stress (Xiao et al. 2025).

Understanding plant responses to combined stress requires careful selection of physiological parameters tailored to the experimental context. Under field conditions, where environmental variables fluctuate dynamically, integrative and non‐invasive measurements of chlorophyll fluorescence are widely employed to assess PSII efficiency and overall stress status (Maxwell and Johnson 2000). In contrast, controlled environment experiments enable more mechanistic dissection of combined stress effects by allowing precise control of temperature, light, humidity, and soil water status. In growth chambers or greenhouses, common parameters such as leaf gas exchange (net photosynthesis and stomatal conductance), stem diameter variation, and leaf water potential are frequently measured to understand physiological limitations under defined stress regimes. Gravimetric phenotyping platforms enable continuous real‐time monitoring of whole‐plant transpiration and growth responses under combined stresses, closely mimicking field scenarios while controlling different variables (Dalal et al. 2020). Moreover, optical measurements combining leaf reflectance indices with chlorophyll fluorescence have been recently developed to track photosynthetic behavior dynamically within controlled environments, providing mechanistic insights into xanthophyll cycle activity and photoprotective responses (Gamon et al. 2019). The choice of parameter thus reflects a balance between ecological relevance and experimental precision. Field‐based chlorophyll fluorescence and temperature‐humidity indices provide rapid stress assessments across heterogeneous environments (Gerhards et al. 2019; Ji et al. 2020), while controlled environment assays enable detailed physiological and molecular analyses underpinning heat tolerance mechanisms. Integrating parameter selection with experimental design enhances the interpretation of plant stress responses and supports breeding efforts aimed at improving crop resilience.

## 4 Biochemical Traits Indicative of Response to Drought and Heat Stress

**FIGURE 2:**
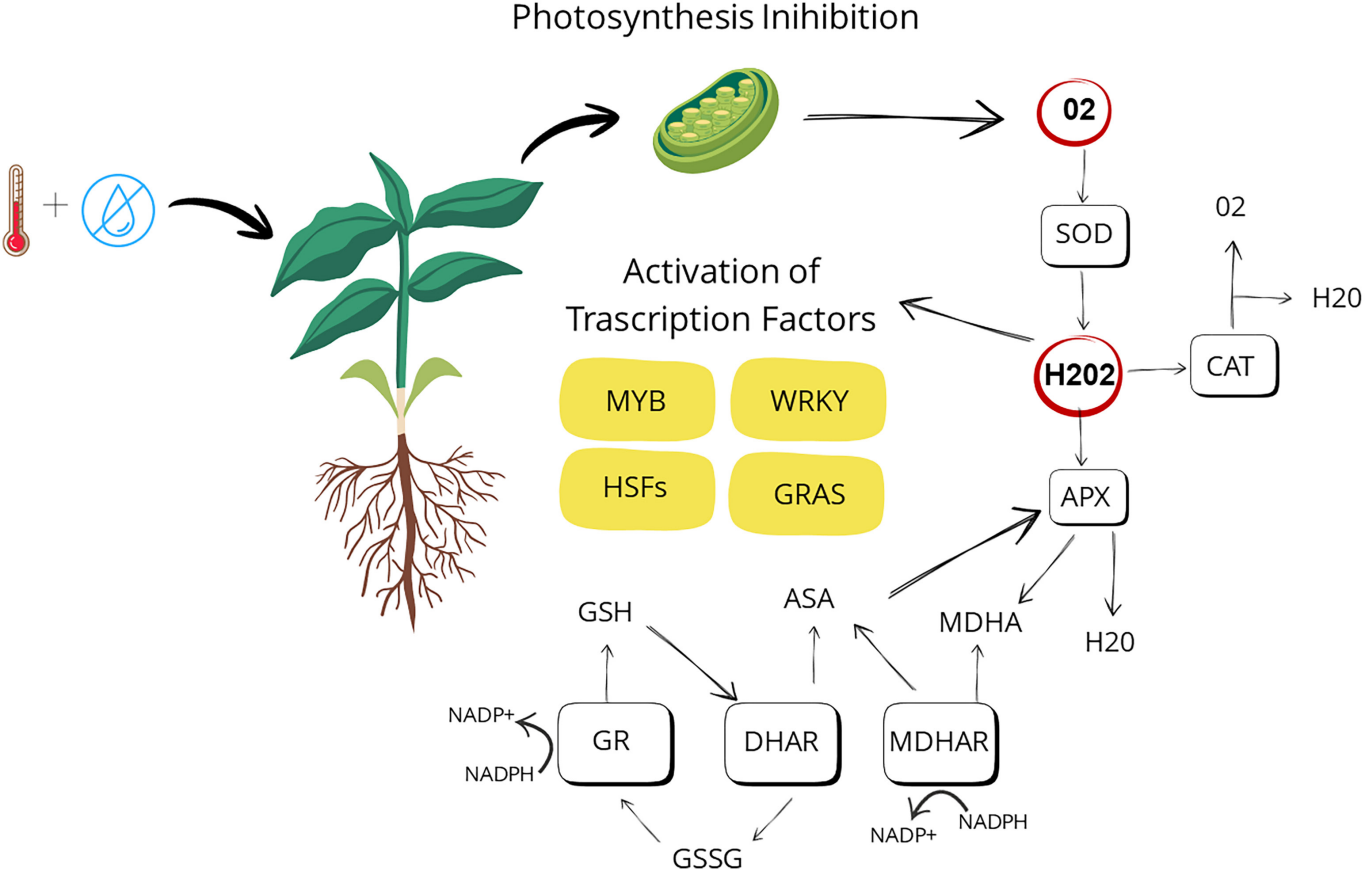
A schematic model of the coordinated antioxidant and transcriptional response triggered by combined heat and drought stress. Inhibition of photosynthesis enhances superoxide production (O_2_^−^), which is dismutated by superoxide dismutase (SOD) to form hydrogen peroxide (H_2_O_2_). This H_2_O_2_ is then detoxified by catalase (CAT) and ascorbate peroxidase (APX). The latter operates alongside the enzymes of the ascorbate–glutathione cycle: Monodehydroascorbate reductase (MDHAR), dehydroascorbate reductase (DHAR), and glutathione reductase (GR). The accumulation of H_2_O_2_ also functions as a signaling molecule that activates stress‐responsive transcription factors, including MYB, WRKY, heat shock factors (HSFs), and GRAS proteins. This modulates the expression of downstream genes under combined abiotic stress.

Photosynthetic pigments—including chlorophyll, carotenes, and xanthophylls (e.g., violaxanthin and zeaxanthin)—serve as key indicators of photosynthetic efficiency and dissipating excess energy (Demmig‐Adams et al. 2020) (Figure 2). Additionally, components of the redox regulatory system are widely used as stress‐monitoring biomarkers. In chloroplasts, the components of the ascorbate–glutathione (Asc‐GSH) cycle are the most dominant, involving ascorbate peroxidase (APX), monodehydroascorbate reductase (MDAR), dehydroascorbate reductase (DHAR), and glutathione reductase (GR), along with the redox states of ascorbate (Asc/DHA), glutathione (GSH/GSSG), and NADPH/NADP^+^ (Foyer and Kunert 2024) (Figure 2). The Asc‐GSH cycle also operates in the cytosol, mitochondria, and peroxisomes (Jimenez et al. 1997). Moreover, in peroxisomes, catalase (CAT) is considered the primary H_2_O_2_ scavenger, though it can also be found in the cytosol, nucleus, and mitochondria (Baker et al. 2023). In the apoplast and the vacuoles, H_2_O_2_ level is maintained by class III peroxidases (PODs) in combination with Asc and phenolics, such as hydroxycinnamic acids and flavonoids; thus, monitoring the levels of these compounds provides valuable insight into the severity of oxidative stress (Ferreres et al. 2011; Takahama 2004). To maintain cell turgor, plants accumulate osmoprotectants, such as proline and sugars; therefore, the analysis of these metabolites can be important in assessing drought response (Vishwakarma et al. 2017).

The reliability of any of these analyses, regardless of the analytical methods employed, depends fundamentally on proper sample collection, processing, and storage. Sampling should be done in a way to minimize mechanical damages and stress to the plants prior to metabolic arrest through rapid cooling or organic solvent fixation. Mechanical injury during leaf excision triggers immediate local production of superoxide and H_2_O_2_ at wound sites (Fraudentali et al. 2023), leading to jasmonic acid (JA) accumulation and oxidative damage. To prevent such artifacts, samples should be flash‐frozen in liquid nitrogen within seconds of collection (Majer et al. 2016). In addition, diurnal variations and sampling time can affect sample integrity. Circadian rhythms also affect both metabolite levels and enzyme activities (Liebelt et al. 2019). Besides, the levels of antioxidants and pro‐oxidants can vary with the plant's age and developmental status. Kanojia et al. (2020) demonstrated that leaf maturation involves upregulation of oxidative stress response genes and stress hormone pathways, coupled with depletion of antioxidative primary metabolites, ultimately reducing tolerance to drought in older leaves. External factors (such as temperature, humidity, and light) also significantly influence biomarker stability and content. Selection of the appropriate growth conditions that address the research question is crucial. Incorporating sufficient biological replicates and proper controls is essential to distinguish genuine stress responses from natural physiological variation (Noctor et al. 2016).

Current methodologies for assessing the above‐mentioned low‐molecular‐weight stress markers encompass diverse approaches with liquid chromatography‐mass spectrometry (LC–MS) being particularly prominent. However, special considerations are crucial when evaluating the redox states of Asc and GSH. Plant leaf cells maintain Asc and GSH at millimolar range, but these levels are dynamically influenced by plant species, growth stage, water and nutrient availability, light duration, intensity and spectrum, as well as the oxidative stress levels (Veljović‐Jovanović et al. 2018). For example, a healthy plant is expected to have a highly reduced ascorbate state (80%–95%) and glutathione state (90%–95%) (Foyer and Noctor 2011). Comprehensive guidelines for measuring Asc and GSH redox states are provided in authoritative reviews (Majer et al. 2016; Noctor et al. 2016), emphasizing fast extraction protocols under dark conditions using metal chelators and low pH, with 1.5% meta‐phosphoric acid containing 1 mM EDTA being optimal. Moreover, reduced glutathione readily oxidizes or forms complexes with vacuolar or apoplastic metals (Keunen et al. 2016), potentially leading to underestimation of glutathione content and its redox state.

As mentioned, changes in antioxidant enzyme activities are recognized as reliable markers of stress responses. Each enzyme necessitates specific conditions aligned with its optimal pH, temperature ranges, cofactor requirements, and stability profile. To achieve accurate results in enzyme assays, it is crucial to optimize extraction protocols for each plant species, considering specific enzyme requirements such as pH, temperature, and stabilizers—e.g., Asc for chloroplastic APX. Proper enzyme assay optimisation must include accounting for blanks and non‐enzymatic kinetics.

Total Antioxidant Capacity (TAC) assays are widely used to evaluate the antioxidative potential of extracts, but variations in the electron acceptors used can lead to inconsistent results. Moreover, some tests may underestimate or overestimate TAC due to residual peroxidase activity or antioxidants reacting with H_2_O_2_ instead of the given pro‐oxidant. Therefore, relying on a single TAC method for ranking extracts is not advisable. However, by using multiple methods and their integration with physiological measurements, individual antioxidant levels and actual ROS content (e.g., measured by electron paramagnetic resonance spectroscopy; Majer et al. 2016) can give a more comprehensive understanding of the antioxidant interactions in tissues under specific conditions.

Interpreting biochemical and physiological traits for assessing stress requires their integration within the complex cellular response network. The adaptive responses can manifest as elevated antioxidant activity, reflecting increased pro‐oxidant production, or as pro‐oxidant accumulation, indicating either enhanced generation or reduced scavenging capacity (Majer et al. 2016). Depending on the context and accompanying physiological data, the obtained changes may indicate initial stress perception, successful acclimation, or protective system failure.

Finally, we should be aware that measuring stress biomarkers from bulk tissue provides only a general indication of cellular status. For more precise assessments, subcellular‐level analysis using compartment‐specific markers is essential. Redox homeostasis is tightly regulated at the organelle level, with chloroplasts, mitochondria, and peroxisomes each maintaining unique redox signatures and antioxidant systems (Foyer and Noctor 2011). Recent advances in fluorescent protein sensors and immunogold labeling techniques allow better spatial resolution of redox processes, revealing significant heterogeneity even within single cell types (Babbar et al. 2021; Exposito‐Rodriguez et al. 2017; Ugalde and Meyer 2025; Vidović et al. 2016). These methods offer better accuracy compared to traditional DAB or NBT staining procedures for measuring H_2_O_2_ and O_2_^•–^, which are not specific and can introduce artefacts (Noctor et al. 2016).

## 5 Molecular Traits Indicative of Response to Drought and Heat Stress

The molecular fingerprints of stress responses in reproductive tissues are highly dynamic and involve genes with diverse functions, ranging from stress sensing and signaling to the activation of transcription factors, heat shock genes (HSGs), transmembrane transporters, sugar‐metabolizing enzymes, and other stress‐related genes. Abiotic stress triggers multiple responsive pathways in reproductive tissues and organs, with gene expression profiles that are often distinct but partially overlapping across the different stress types encountered under field conditions. Recent advances in high‐throughput sequencing technologies and bioinformatic pipelines have facilitated the dissection of molecular mechanisms underlying stress tolerance, particularly during the reproductive stage (Kaundal et al. 2021). However, elucidating stress tolerance mechanisms at this critical developmental stage requires meticulous experimental design, careful tissue selection, and comprehensive data analysis—encompassing the identification of regulatory genes and pathways, their functional characterization, and rigorous experimental validation (Kaur et al. 2023). In this review, we provide a concise overview of key molecular pathways and genes activated in response to abiotic stress imposed during the reproductive stage.

### 5.1 Transcription Factors Involved in Drought and Heat Stress Tolerance

Transcription factors (TFs) are central regulators of gene expression during reproductive development, modulating stress‐responsive pathways and developmental timing to enhance resilience under stress. Transcriptomic profiling of rice florets under drought identified over 1000 responsive genes, including 83 TFs—among them, MIKCc‐type MADS‐box genes such as *OsMADS18*, which promotes early flowering (Fornara et al. 2004; Jin et al. 2013; Table 1).

**TABLE 1:** Stress‐responsive genes involved in reproductive‐stage abiotic tolerance across crops.

Gene	Gene family/type	Stress	Crop	Author(s)
Transcription factors involved in abiotic stress tolerance				
*OsMADS18*	MIKCc‐type MADS‐box TF	Heat	Rice	Fornara et al. 2004
*OsHsfA2a, OsHsfA2d, OsHsfB2a*	Heat Shock Factors (HSFs)	Heat	Rice	Chauhan et al. 2011
*HsfA2*	Heat Shock Factor	Heat	Tomato	Fragkostefanakis et al. 2016
Calcium and ROS signaling‐related genes				
*RbohH, RbohJ*	NADPH Oxidases	Heat	Rapeseed	Lohani et al. 2021
*SlAPX3*	APX	Heat	Tomato	Frank et al. 2009
*BnaA06g04380D, BnaC05g05550D, BnaA01g32160D*, *BnaC01g39080D*	APX	Heat	Rapeseed	Lohani et al. 2021
*BnaCnng14420D*	Cyclic nucleotide‐gated ion channel (CNGC)	Heat	Rapeseed	Lohani et al. 2021
Defense‐related regulation genes				
*HvHspc70‐4*, *HvHspc70‐5a*, *HvHspc70‐5b*, *HvHsp90‐1*, *HvHsp100‐1*, *HvHsp100‐2*	HSP	Heat	Barley	Chaudhary et al. 2019
*Os01g62290, Os05g38530, Os03g11910*	HSP	Heat	Rice	Sarkar et al. 2013
*LEA3*, *LEA14*, *LEA19*	LEA proteins	Drought	Rice	Kaur et al. 2023
*CESA6, BnaA02g34360D*	Cellulose synthase	Heat	Rapeseed	Lohani et al. 2021
*OsWR2*	Wax synthesis regulator	Heat	Rice	Kan et al. 2022
TT2	Thermo Tolerance regulator	Heat	Rice	Kan et al. 2022
*NAT1*	Transcriptional repressor	Heat	Rice	Lu et al. 2025
*CER1, CER1L*	Wax biosynthesis genes	Heat	Rice	Lu et al. 2025
*cwINV*	Cell Wall Invertase	Drought	Wheat	Koonjul et al. 2005
Secondary metabolite biosynthesis genes				
*GRMZM5G872256, GRMZM2G165919*	Galactinol synthase	Drought	Maize	Kakumanu et al. 2012
*GRMZM2G089713, GRMZM2G318780*	Sucrose synthase	Drought	Maize	Kakumanu et al. 2012
*Os02g41630, Os08g14760, Os09g04050, Os10g36848*	Lignin synthesis	Heat	Rice	Cai et al. 2020
*Os11g32650, Os11g02440, Os12g02370, Os04g56700, Os01g44260*	Flavonoids synthesis	Heat	Rice	Cai et al. 2020
Biosynthesis of phytohormones				
*NCED3*	ABA biosynthesis	Heat & drought	Rice	Ma et al. 2024
*OsYUCCA*	Auxin biosynthesis	Drought	Rice	Duan et al. 2023
*GhAOC*2	JA synthesis	Heat	Cotton	Khan et al. 2020

Comparative analysis of drought‐tolerant and sensitive rice cultivars revealed nearly 800 differentially expressed TFs (Kaur et al. 2023). Tolerant genotypes showed enrichment of regulatory families including bHLH, NAC, ERF, WRKY, MYB, and GRAS, with GO terms linked to transcriptional regulation and water deprivation. In contrast, sensitive cultivars primarily activate structural pathways such as translation and ribosome biogenesis.

Heat stress in rapeseed similarly disrupted reproductive development. Enriched GO terms in stressed pollen and pistils included “response to heat” and “protein folding,” while downregulated genes were associated with ion transport, carbohydrate metabolism, and cuticle formation (Lohani et al. 2021). These patterns suggest conserved transcriptional reprogramming across species.

TF families consistently implicated in stress include DREB2A, AP2/ERF, bZIP, WRKY, MYB, and HSFs (Figure 2). Notably, MYB TFs are downregulated in heat‐stressed pistils but upregulated in maize pollen (Begcy et al. 2019). Heat Shock Factors such as OsHsfA2a and HsfA2 (rice and tomato) are key regulators of pollen viability under heat stress (Chauhan et al. 2011; Fragkostefanakis et al. 2016).

### 5.2 Calcium and ROS Signaling‐Related Genes

Heat stress can increase cyclic nucleotides (cAMP, cGMP), which activate calcium ion channels (cyclic nucleotide‐gated channels, CNGCs), triggering Ca^2+^ influx in pollen grains (Gao et al. 2012; Tunc‐Ozdemir et al. 2013). This influx initiates heat shock response (HSR) by activating HSFs, thereby protecting plants from heat stress. Calcium ions are critical for regulating pollen germination and polar growth, establishing gradients at the germination sites that drive pollen tube elongation (Ge et al. 2007; Qian et al. 2025). However, differential expression of Ca^2+^ signaling genes under stress can disrupt pollen tube growth and fertilization (Lohani et al. 2021).

Calcium signaling is tightly integrated with ROS dynamics, forming a coordinated network that regulates stress responses and reproductive success. ROS play dual roles in reproduction, acting as both damaging agents and essential signaling molecules. During microspore maturation under heat stress, multiple APX enzyme isoforms detoxify ROS while modulating H_2_O_2_‐based signaling, thereby contributing to pollen tube growth and thermotolerance (Frank et al. 2009; Potocký et al. 2007).

In *Brassica napus* , heat stress impairs pollen and pistil function through misregulation of ROS‐related genes. Downregulation of NADPH oxidases (*RbohH* and *RbohJ*), which generate ROS, occurs alongside upregulation of *GALACTINOL SYNTHASE 1* and *RAFFINOSE SYNTHASE* family, indicating a compensatory antioxidant response through raffinose family oligosaccharide biosynthesis (Lohani et al. 2021; Salvi et al. 2018). These findings highlight a fine‐tuned gene‐regulatory balance between ROS production, signaling, and scavenging that underpins reproductive resilience. Under normal conditions, ROS promotes pollen germination. However, excessive ROS damages pollen membranes (Das et al. 2014) and can induce dormancy in some pollen grains under heat stress, with subsequent germination occurring at lower temperatures, allowing pollen to avoid peak heat stress (Rutley et al. 2022).

### 5.3 Defense‐Related Regulation Genes

Defense‐related regulation genes, particularly *HSP*s, play an important role in maintaining cellular integrity and reproductive viability under abiotic stress. HSPs act as ATP‐dependent molecular chaperones, refolding denatured proteins and preventing aggregation. In barley, genome‐wide expression analysis revealed significant upregulation of Hsp70, Hsp90, and Hsp100 family members in reproductive tissues under heat stress, with additional expression under drought, salinity, and heavy metal stress (Chaudhary et al. 2019). Similar observations have been reported in rice (Sarkar et al. 2013; Table 1).

Late embryogenesis abundant (LEA) proteins, widely recognized for their role in drought tolerance, stabilize cellular structures and preserve protein function under water deficit. Although most studies focus on vegetative tissues, transcriptomic and proteomic data suggest their contribution to reproductive‐stage stress protection, especially in drought‐tolerant genotypes (Kaur et al. 2023; Table 1).

Heat stress adaptation in pollen involves changes in cell wall composition and cuticle integrity. Downregulation of cellulose synthase genes reduces pollen wall flexibility, while wax biosynthesis regulators maintain cuticle function under elevated temperatures (Lohani et al. 2021; Kan et al. 2022). Transcriptional repressors have also been identified that modulate wax biosynthesis and starch accumulation in pollen, both crucial for maintaining fertility under heat stress (Lu et al. 2025).

Genes associated with anther development—including those regulating pollen wall formation and tapetum degeneration—are differentially expressed under drought (Jin et al. 2013). In wheat, drought reduces the activity of cell wall and vacuolar invertases, halting pollen development due to impaired carbohydrate availability (Koonjul et al. 2005).

Similarly, in maize (Liu et al. 2022), drought during the stamen elongation phase suppresses auxin‐related genes (e.g., small auxin up RNA, *SAUR*), photosynthesis‐related genes, and cell elongation‐related genes such as expansin, aquaporin, and xyloglucan endotransglucosylase, leading to slow silk elongation and increased pollen sterility (Danilevskaya et al. 2019). These findings suggest that improving drought resilience in reproductive tissues requires crop‐specific strategies.

### 5.4 Secondary Metabolite Biosynthesis Genes

Secondary metabolites play a major role in protecting reproductive tissues from stress. Flavonoids, in particular, regulate ROS dynamics, influencing pollen germination, tube growth, and structural integrity (Qian et al. 2025). In rice, differential expression of genes involved in phenylpropanoid, terpenoid, and lignin/lignan biosynthesis was observed under drought and heat stress (Kaur et al. 2023).

Transcriptomic analysis of the heat‐tolerant rice germplasm SDWG005 revealed that reproductive‐stage heat stress (in spikelets) significantly upregulated genes in lignin and flavonoid biosynthesis pathways, which are strongly linked to thermotolerance during reproductive development (Cai et al. 2020).

Moreover, RNA‐Seq analysis of maize ovary tissue under drought identified upregulation of genes in the raffinose oligosaccharide pathway, including galactinol synthase genes, suggesting ROS scavenging functions (Kakumanu et al. 2012).

### 5.5 Biosynthesis of Phytohormones

The abscisic acid (ABA) pathway is a central regulator of reproductive‐stage stress responses, particularly under drought (Nambara and Marion‐Poll 2005). Comparative transcriptomic studies in rice cultivars revealed contrasting ABA biosynthesis gene expression, with tolerant lines upregulating key components and sensitive ones showing widespread downregulation (Kaur et al. 2023). Elevated ABA biosynthesis in reproductive meristems under heat and drought further underscores its role in inflorescence‐specific stress signaling (Ma et al. 2024). Elevated ABA in anthers further promotes ROS buildup and premature cell death (Zhao et al. 2023). In maize, drought triggers embryo abortion by activating ABA‐ and senescence‐related signaling post‐fertilization (Kakumanu et al. 2012).

Other phytohormones, including brassinosteroids (BRs), auxins, cytokinins, gibberellins (GAs), JA, and salicylic acid, also contribute to reproductive resilience. BRs support thermotolerance and cellular homeostasis, while drought suppresses auxin biosynthesis, impairing floral organ development (Duan et al. 2023). GA signaling regulates anther dehiscence, and its suppression under heat stress leads to increased DELLA protein levels, reduced JA signaling, and male sterility (Hu et al. 2008; Huang et al. 2022; Khan et al. 2023). JA is similarly important for anther dehiscence, with reduced JA levels under heat stress correlating with ROS accumulation and impaired fertility (Peng et al. 2013).

### 5.6 Availability of Soluble Carbohydrates

Carbohydrate metabolism supports reproductive success under abiotic stress (Qian et al. 2025). Heat stress reduces soluble carbohydrate availability and invertase activity in maize pistils, limiting the energy supply needed for pollen tube growth (Wang et al. 2024). Also, stigma receptivity is reduced due to oxidative stress (Qian et al. 2025).

Drought stress also induces carbohydrate deficits. In rice, reduced pollen number and germination under drought are linked to sugar starvation pathways (Li et al. 2015). In maize and tomato, increased carbohydrate biosynthesis and flavonoid levels have been associated with improved anther development (Muhlemann et al. 2018). However, drought induces downregulation of *cwINV* and vacuolar invertase genes, and sugar transport genes in maize pistils, reducing sucrose uptake and allocation (Ren et al. 2020). As carbohydrate resources to the ovule become scarce, genes encoding ribosome‐inactivating protein and phospholipase D are activated, triggering early senescence. Importantly, sucrose supplementation to flowers restored yield under drought, underscoring the major role of sugar metabolism in reproductive resilience (Zinselmeier et al. 1995).

### 5.7 Exploitation of Genes That Can Be Used in Creating Climate‐Smart Crops

To address the severe impact of high temperatures on crop productivity, breeders are developing varieties with enhanced heat tolerance. In rice, a loss‐of‐function allele of *THERMOTOLERANCE 2* (*TT2*) enhances wax biosynthesis under stress by stabilizing *OsWR2* expression, improving thermal resilience (Kan et al. 2022). Additionally, NAT1, a heat‐induced nuclear repressor, downregulates *bHLH110*, modulating *ECERIFERUM1 (CER1)* and *CER1‐*like *(CER1L)*, which are essential for wax very‐long‐chain alkane synthesis (Lu et al. 2025). NAT1 also regulates starch accumulation in mature pollen, influencing fertility. Initial field evaluations suggested that *NAT1*‐edited lines can achieve higher seed set and grain yield; nevertheless, this approach should be considered preliminary, as broader multi‐location and multi‐season testing is still needed to confirm its robustness.

Genotypic variation in pollen traits also offers opportunities for selection. In tomato, Paupière et al. (2017) identified heat‐tolerant genotypes based on pollen characteristics, while similar findings have been reported in lentils, soybeans, and cotton (Qian et al. 2025).

In rice, introgression of *qHTH5* from *Oryza rufipogon* enhanced the seed‐setting rate by ~30% under heat stress at the heading stage, which showed the potential of using this common wild rice as a genetic resource for increasing heat tolerance (Cao et al. 2022). Similarly, the EMF20 introgression line derived from *O. officinalis* advanced flowering to cooler early‐morning hours, reducing heat‐induced spikelet sterility and demonstrating the potential of wild‐rice EMF (Early Morning Flowering) alleles to support breeding for resilience in warming climates (Hirabayashi et al. 2015; Ishimaru et al. 2010). In addition, the knockout of *OsRbohB* lowered ROS accumulation and improved cellular integrity, floral development, and starch synthesis under heat stress, identifying *OsRbohB* as a negative regulator of heat responses and a candidate target for developing heat‐tolerant rice through precise genome editing (Qian et al. 2025).

Beyond heat tolerance, genes associated with drought resilience are equally important. For instance, the group I LEA gene *OsEm1* is strongly induced by drought and ABA during reproductive stages, and its overexpression enhances drought tolerance, making it a promising candidate for genetic engineering (Yu et al. 2016).

To mitigate the impacts of climate change, it is crucial to exploit natural genetic variation and apply targeted genome editing on heat‐ and drought‐related genes, particularly those associated with reproductive development in order to develop crops with enhanced reproductive resilience.

## 6 Multi‐Omics Techniques for Monitoring Responses to Drought and Heat Stress

Over the past 20 years, various strategies have been developed to identify new stress markers in crops, including genomics, epigenomics, transcriptomics, proteomics, ionomics, metabolomics, and phenomics (Albert et al. 2018; Roychowdhury et al. 2023). Recent reviews emphasize that such “pan‐omics” or multi‐omics strategies, when combined with advanced phenotyping and breeding, are central to developing climate‐resilient crops (Chaturvedi et al. 2024; Weckwerth et al. 2020). Multi‐omics has also emerged as a powerful approach for dissecting reproductive resilience and pollen thermotolerance (Chaturvedi et al. 2021). Within this spectrum, transcript‐based approaches are still the most widely applied, including precise methods such as quantitative reverse transcription PCR (qRT‐PCR) and microarrays, while RNA sequencing (RNA‐Seq) has become the most widely adopted method, enabling unbiased analysis of thousands of RNA transcripts in parallel, including noncoding and small RNAs (Ali et al. 2022; Arbona et al. 2013; Chaudhary et al. 2019). In parallel, phenomics now ranges from automated imaging platforms in controlled environments to drone‐ and satellite‐based thermal sensing, with recent validation studies highlighting the importance of rigorous calibration of canopy temperature indices and computer vision pipelines against physiological measurements (Ndlovu et al. 2024; Putra Hernanda et al. 2024; Renó et al. 2024).

A major challenge in –*omics* approaches is the limited availability and standardization of data. Although next‐generation sequencing (NGS), processed expression data, proteomics, metabolomics, and protein structures are routinely deposited in global repositories (NCBI Resource Coordinators 2018; wwPDB consortium 2019), datasets often remain incomplete, poorly annotated, or lack standardized metadata. This is particularly problematic for stress biology, where integrative analyses depend on consistent descriptions of treatments, developmental stages, and sampling protocols (Jamil et al. 2020; Krantz et al. 2021). Because plant phenotypes can exhibit significant variations in response to environmental changes, it is imperative to establish and follow consistent standards for metadata descriptions, particularly regarding growth conditions, stress regimes, and phenotyping protocols.

Despite their widespread use, all –*omics* approaches have limitations, and results must be interpreted with caution. For example, whole‐genome sequencing (WGS) may introduce biases due to uneven coverage across genomic regions, particularly those enriched in repetitive elements or high GC content. High‐accuracy sequencing technologies combined with robust bioinformatics tools can help mitigate these issues (Mardis 2008). In plants, additional complexities such as gene duplication, gene loss, and functional redundancy among paralogs further complicate analysis (Jha et al. 2021). Inclusion of diverse populations is also critical for capturing broad genetic variation and avoiding biases that could mislead downstream interpretations (Fatumo et al. 2022). Approaches that couple multi‐omics with genomic selection and germplasm diversity highlight how data integration can lead to precision breeding (Weckwerth et al. 2020; Zenda et al. 2021).

High‐quality RNA remains a prerequisite for reliable and reproducible RNAseq data. Secondary metabolites such as phenolics, pigments, and carbohydrates may interfere with RNA extraction and quantification protocols (Brescia and Banks 2009; Wang and Stegemann 2010). In drought and desiccation studies, dehydrated tissues accumulate osmoprotectants and polysaccharides that complicate RNA isolation (Meng and Feldman 2010; Valenzuela‐Avendaño et al. 2005). Polyphenols can be removed using polyvinylpyrrolidone (PVP), which binds phenolic hydroxyl groups through strong hydrogen bonding (Japelaghi et al. 2011). Oxidative enzymes such as polyphenol oxidases and PODs can further complicate RNA extraction by generating quinones that modify nucleic acids (Wurtmann and Wolin 2009). Robust extraction protocols using TRIzol and cetyltrimethylammonium bromide (CTAB) have been optimized for recalcitrant plant material (Souza‐Perera et al. 2016; Vidović and Ćuković 2020).

Recent advancements in quantitative proteomics provided deeper insight into the molecular mechanisms of stress responses. As an example, a proteomic approach has been used to unravel the mechanisms controlling pollen heat stress response and ethylene‐mediated pollen thermotolerance in tomato developing pollen grains (Jegadeesan et al. 2018). Protein extraction remains a critical step, complicated by interfering compounds such as polysaccharides and polyphenols that can oxidize or aggregate proteins (Kallinich et al. 2018; Pierpoint 2004; Saravanan and Rose 2004). Another challenge of proteomic studies is their focus on whole tissues, which limits the characterization of low‐abundance proteins crucial for stress tolerance. Therefore, high‐throughput single‐cell or cell‐type‐specific proteomics is needed to refine stress marker identification (Balasubramanian et al. 2021). In addition, proteomic analyses should evaluate not only protein abundance but also post‐translational modifications (phosphorylation, glycosylation) and protein–protein interactions. Protein annotation and functional information in public databases are steadily improving, which facilitates multi‐omics integration.

Metabolomics faces distinct challenges due to the vast diversity of metabolites with varying structures and properties. A single Arabidopsis accession, for example, may contain more than 5000 metabolites, many of which remain uncharacterized. Unlike transcriptomics, there is no universal method capable of capturing the full metabolome. Extraction, fractionation and detection techniques must therefore be optimized for enhancing coverage (Kim and Verpoorte 2010). Most profiling approaches are targeted and cover metabolites with similar properties, thus covering only a small fraction of the metabolome, while non‐targeted fingerprinting techniques such as nuclear magnetic resonance and Fourier‐Transform Infrared spectroscopy offer broader but less specific insights. Broader coverage can be achieved by combining separation techniques such as gas chromatography or LC with MS and by integrating metabolomic profiles with genomic and phenotypic information in breeding programs (Weckwerth et al. 2020).

To gain a comprehensive understanding of plant stress responses and identify relevant molecular traits, integration of transcriptomics, proteomics, and metabolomics data is essential. Such multi‐omics integration enables a comprehensive understanding of the complex biological processes that underlie a plant's response to abiotic stress and identifies key molecular players involved in tolerance responses. This holistic multi‐omics framework provides a detailed overview of the development, functions, and interrelationships of cells, tissues, or organisms by characterizing and quantifying their biomolecules through high‐performance methodologies (Chaturvedi et al. 2024; Jha et al. 2021; Mosa et al. 2017; Weckwerth et al. 2020).

A common focus in transcriptomics and proteomics is the variable correlation between mRNA abundance and the corresponding protein levels. Numerous studies have demonstrated weak correlations between these two factors (Fernie and Stitt 2012), especially during developmental transitions (Ross et al. 2021) and under stress conditions (Vogel and Marcotte 2012). For example, an integrative study on the resurrection plant *Ramonda serbica* , which combined transcriptomics, proteomics, metabolite and photosynthetic measurements, revealed a weak correlation between DEGs and differentially abundant proteins (DAPs) in hydrated versus desiccated leaves (Vidović et al. 2022). Similarly, in tomato pollen, the coupling of transcriptome and proteome adaptation during development and heat stress revealed extensive stage‐specific regulation and only partial concordance between RNA and protein changes (Keller et al. 2018). Such discrepancies may partly reflect the limited sensitivity of current sequencing and proteomic technologies, but they also indicate that translational control and post‐translational modifications are crucial components of stress responses that must be captured by multi‐omics approaches, Ross et al. (2021).

## 7 Conclusion and Future Perspectives

**FIGURE 3:**
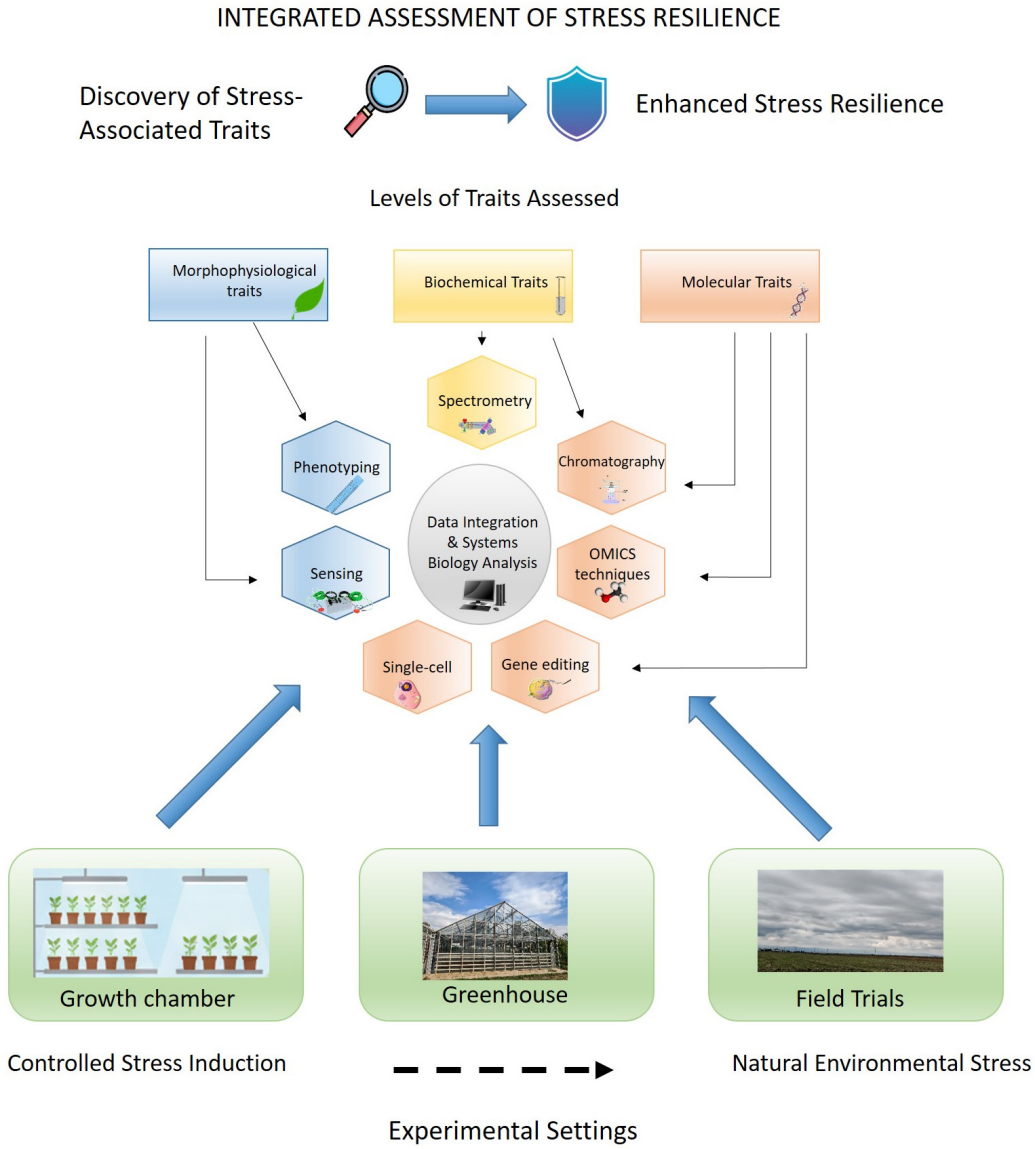
Multi‐level approaches for assessing morphophysiological, biochemical and molecular traits of abiotic stress tolerance. This framework illustrates how morphophysiological, biochemical, and molecular traits are assessed to identify stress‐associated features and enhance plant resilience. Traits are evaluated using diverse techniques, including phenotyping, sensing, spectrometry, chromatography, omics approaches, single‐cell analysis, and gene editing, and integrated through systems biology analysis. Evaluations can be carried out under varying levels of control, ranging from growth chambers (precise stress induction) to greenhouses (semi‐controlled environments) and field trials (natural stress conditions). These complementary settings allow trait assessment across a gradient of experimental control.

In this review, key methodologies for assessing crop resilience to single and combined abiotic stresses were discussed (Figure 3). Over the last decade, significant progress has also been made in the development of various *‐omics* techniques, including approaches that achieve spatial resolution at the single‐cell level (Balasubramanian et al. 2024; Cho et al. 2025; Zhu et al. 2025). Single‐cell multi‐omics is emerging as an essential tool for uncovering the molecular mechanisms underlying stress tolerance occurring within individual plant cells, providing cell‐type‐resolved views of gene expression, chromatin state, and metabolic activity that can be directly linked to developmental and environmental cues (Jha et al. 2021; Zhu et al. 2025). In complementation to single‐cell transcriptomics, spatial transcriptomics reveals the specific location of gene expression profiles across the selected tissue section, facilitating precise identification of genes and gene regulatory networks that can be used for enhancing crop yield, quality, and resilience (Hu et al. 2024; Jain 2024). Methodological advances in nuclei isolation and snRNA‐Seq workflows, including improved protocols for leaf tissues, now make it feasible to apply single‐cell omics in a broader range of crop species and stress scenarios (Madrid et al. 2025; Sunaga‐Franze et al. 2021). Despite its promise, achieving spatial resolution in plants remains technically challenging, as it requires efficient isolation of cells (protoplasts) or nuclei while preserving their integrity (Grones et al. 2024). Additionally, the resulting data must be carefully contextualized within functional annotations of cell types. A further limitation is the lack of validated cell‐type‐specific marker genes in non‐model species, which limits the functional characterization of cell types based solely on transcriptomic data. However, investigating the molecular changes that occur within single cells and understanding how these alterations are communicated across tissues and between organs will be crucial for deciphering how plants perceive and acclimate to fluctuating environmental factors (Jha et al. 2021; Zhu et al. 2025). Integrating these high‐resolution approaches with systems‐level multi‐omics and advanced phenotyping will potentially enable the identification of biomarkers of reproductive resilience and support the breeding of climate‐smart crops.

## Author Contributions

C.B., M.V., A.R., A.B., S.F., M.M.R. contributed to writing the initial draft, conceptualization, review, and editing of the manuscript. All authors contributed significantly and approved the final version for publication.

## Funding

This project was supported by the Agritech National Research Center and received funding from the European Union Next‐Generation EU (Piano Nazionale di Ripresa e Resilienza (PNRR)—Missione 4 Componente 2, Investimento 1.4–D.D. 1032 17/06/2022, CN00000022).

## Conflicts of Interest

The authors declare no conflicts of interest.

## Data Availability

The authors have nothing to report.
